# Development of Transcriptomic Markers for Population Analysis Using Restriction Site Associated RNA Sequencing (RARseq)

**DOI:** 10.1371/journal.pone.0134855

**Published:** 2015-08-04

**Authors:** Magdy S. Alabady, Willie L. Rogers, Russell L. Malmberg

**Affiliations:** Department of Plant Biology, University of Georgia, Athens, GA, 30604, United States of America; Clemson University, UNITED STATES

## Abstract

We describe restriction site associated RNA sequencing (RARseq), an RNAseq-based genotype by sequencing (GBS) method. It includes the construction of RNAseq libraries from double stranded cDNA digested with selected restriction enzymes. To test this, we constructed six single- and six-dual-digested RARseq libraries from six F2 pitcher plant individuals and sequenced them on a half of a Miseq run. On average, the de novo approach of population genome analysis detected 544 and 570 RNA SNPs, whereas the reference transcriptome-based approach revealed an average of 1907 and 1876 RNA SNPs per individual, from single- and dual-digested RARseq data, respectively. The average numbers of RNA SNPs and alleles per loci are 1.89 and 2.17, respectively. Our results suggest that the RARseq protocol allows good depth of coverage per loci for detecting RNA SNPs and polymorphic loci for population genomics and mapping analyses. In non-model systems where complete genomes sequences are not always available, RARseq data can be analyzed in reference to the transcriptome. In addition to enriching for functional markers, this method may prove particularly useful in organisms where the genomes are not favorable for DNA GBS.

## Introduction

Next generation sequencing (NGS) technologies make it possible to perform genome-wide genotyping in hundreds of individuals simultaneously. Several genome-wide genotyping methods using NGS have been developed and proven efficient (reviewed in [1].) These methods can be classified into two types: restriction digestion- and target enrichment-based methods. The main concept of both methods is that a reduction of genome complexity by restriction digestion or target enrichment is necessary to assure sufficient overlap across individuals in the sequence coverage of genomes, especially for the large genomes. NGS methods that deploy restriction digestions include, restriction-site DNA associated sequencing (RADseq)[2] and genotyping by sequencing (GBS)[3]. Both RADseq and GBS methods are similar in the concept, but different in their barcoding and multiplexing modules. Target enrichment methods include SNP discovery from standard RNAseq data [4,5] and from target sequence capture data [6–8]. Conceptually, Genotyping-by-sequencing methods include obtaining whole genomic DNA samples, digesting using specific restriction enzymes then collecting a size range of fragments, or otherwise enriching for particular sequences; these are then uniquely barcoded, multiplexed, and sequenced. In general, restriction-based methods yield genome-wide, unbiased markers, whereas the opposite is true in the target enrichment-based methods. While this method offers many benefits, it also comes with a host of limitations. Organisms with large, highly methylated genomes present a unique challenge when using GBS. Traditional GBS methodology in these organisms can create a massive amount of data consisting largely of non-genic sequences. This leads to shallow coverage over biological significant SNPs and a lower probability of a match of a given sequence across samples than would be desirable. Using standard RNAseq as a genotyping method, on the other hand, is challenged by the presence of alternative transcripts, which make it difficult to infer genotypes.

Here we present a Restriction Site Associated RNAseq (RARseq) approach for genome-wide marker development. In this approach, we utilize mRNA transcriptome in lieu of genomic DNA in the GBS/RADseq protocol. To test the method, we use plants of the genus *Sarracenia* (pitcher plants) which have a genome 25% large than human or maize [9]. We assess the application of RARseq in population genomic analysis in a panel of six F2 individuals generated from the cross *S*. *psittacina* X *S*. *purpurea*. Our results suggest that RARseq is an efficient method for genotyping and population genomic analysis.

## Results

### In silico restriction digestion and RARseq sequencing

We used 454 mRNA transcriptomic sequences generated from *S*. *psittacina* and *S*. *purpurea* [8] to identify the suitable restriction enzymes. The sequences consisted of 9.43Mb in 15,447 reads (%AT = 58.18), and 9.43Mb in 12,306 reads (%AT = 57.78) from *S*. *psittacina* and *S*. *purpurea*, respectively. We used the fuzznuc program from the EMBOSS package [10] to search for the restriction sites patterns in transcriptomes. No mismatches were allowed in the restriction site patterns and target fragment size ranged from 150 to 700bp. The number of dual digested fragments ranged from 61 using *TasI–SbfI* to 5193 using *MseI–BstYI* (Table 1.) In this report, we used single and dual digestions using *MseI* and *MseI-StyI*, respectively, to generate RARseq libraries from six F2 individuals. The 12 libraries were pooled and sequenced on the Illumina MiSeq platform. A total of total of 3,677,390 RARseq reads were obtained from the six F2 individuals. Among these reads, 1,349,116 reads with *MseI* tags (MseI RARtags) and 1,840,270 *MseI-StyI* RARtags were identified. The average number of RARtags per sample is 224852.7 and 306711.8 in the single- and dual-digestion, respectively (Table 2.)

**Table 1:** In silico restriction digestion of the pitcher transcriptome to select restriction enzyme combinations for generating cDNA fragments suitable for RARseq libraries. The target fragment length ranged from 150bp to 700bp in all digestions.

Enzyme combination	Fuzznuc search pattern (Mismatch = 0)	Reported sequences	Reported hit counts
MseI—BstYI	TTAAN(145,700)[AG]GATC[CT]	5193	14896
MspI—PstI	CCGGN(145,700)CTGCAG	1033	1968
MaeII—ApoI	ACGTN(145,700)[AG]AATT[CT]	4614	8176
EcoRI—MseI	GAATTCN(145,700)TTAA	2247	3041
TasI—SbfI	AATTN(145,700)CCTGCAGG	61	189
Sau3A—BstYI	GATCN(145,700)[AG]GATC[CT]	4530	13693
MseI—AflIII	TTAAN(145,700)AC[AG][CT]GT	2737	7298
BstYI—AflIII	[AG]GATC[CT]N(145,700)AC[AG][CT]GT	1179	1681
MseI—StyI	TTAAN(145,700)CC[AT][AT]GG	4643	13280

**Table 2:** Results summary of the STACKS’ population genome analysis using de novo approach. Numbers in parenthesis are the results after applying STACKS correction module to make population-based correction.

F2 individual	MseI RARseq				Mse1-Styl RARseq			
	Number of RARtags	Unique Stacks	Polymorphic Loci	SNPs	Number of RARtags	Unique Stacks	Polymorphic Loci	SNPs
F2-3	108241	1794 (1701)	211 (196)	378 (327)	303222	1929 (1817)	293 (256)	561 (467)
F2-4	186507	1049 (978)	162 (142)	299 (258)	353812	1727 (1639)	269 (240)	494 (418)
F2-6	257352	2537 (2417)	345 (319)	629 (562)	247579	2668 (2533)	468 (424)	848 (739)
F2-7	208467	2424 (2315)	389 (369)	678 (615)	326714	2049 (1930)	329 (293)	623 (531)
F2-8	300259	3143 (2994)	429 (397)	790 (702)	303183	1831 (1717)	292 (265)	526 (452)
F2-9	288290	2058 (1928)	266 (238)	490 (423)	305761	1682 (1551)	273 (233)	491 (387)
Average	224852.7	2167.5 (2055.5)	300.3 (276.8)	544 (481.2)	306711.8	1981 (1868.5)	320.7 (285.2)	590 (499)

### Population analysis of RARseq data

**Fig 1:**
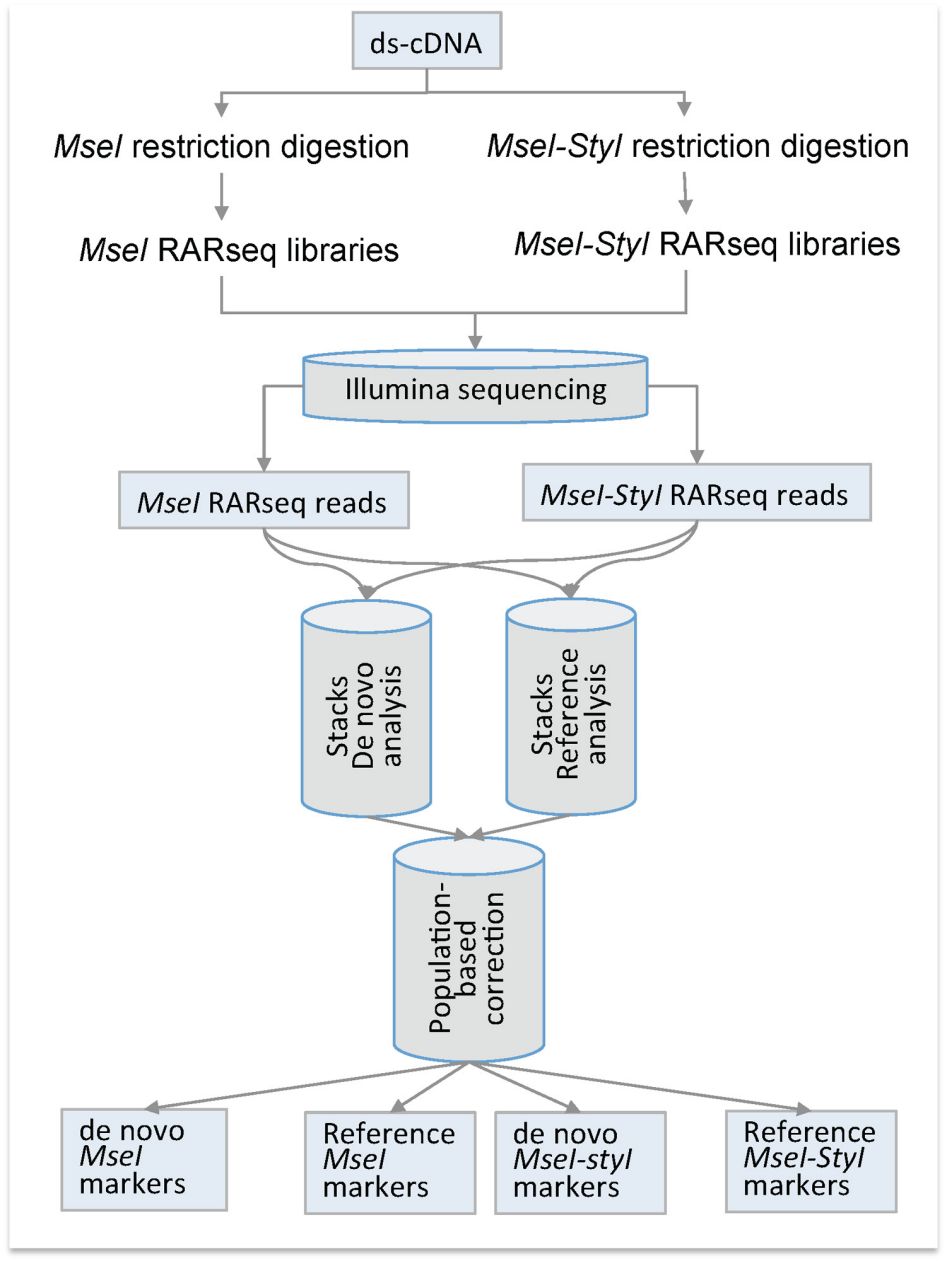
RARseq protocol. The flowchart illustrates workflow and analysis guidelines of the RARseq method.

We used the population genomic analysis in the Stacks package [11] to analyze both *MseI* and *MseI-StyI* RARseq sequencing data. Both de novo and reference approaches were used. For the reference approach, we used a customized transcriptome (see materials and methods) as a reference. The Stacks’ correction model was applied on the analysis results. The outcomes of these analyses are two sets of markers (polymorphic loci and SNPs) from the *MseI* RARseq data and from the *MseI-StyI* RARseq data. Hereafter we will refer to these sets of markers as following: de novo *MseI* markers, reference *MseI* markers, de novo *MseI-StyI* markers, and reference *MseI-StyI* markers (Fig 1).

### Polymorphic markers from single- and dual-digested cDNA

In the de novo analysis, the average number of unique stacks is higher in the *MseI* (2167.5) than in *MseI-StyI* (1981) data. In spite of that, *MseI-StyI* RARseq data showed slightly higher level of detected polymorphic loci and SNPs (Table 2.) In average, 80.9% and 80.4% of *MseI* and *MseI-StyI* RARseq reads, respectively, were mapped to the custom transcriptome using Bowtie [12]. In reference to the transcriptome, the numbers of unique stacks, polymorphic loci, and SNPs are higher in the *Mse*I than in *MseI-Styl* RARseq data (Table 3.) As shown in Tables 2 and 3, both reference *MseI* and *MseI-StyI* data resulted in significantly higher unique stacks, polymorphic loci, and SNPs comparing to the corresponding de novo data. The average number of SNPs that were detected using de novo and reference analyses of *MseI* RARseq are 1907.7 and 544, respectively (about a 3.5 fold change.) In case of the *MseI-StyI* RARseq, the average number of de novo detected SNPs is 590.5, whereas, in average, 1861 SNPs were detected in reference to the transcriptome (about a 3.2 fold change.) Despite the small size of the test population, using STACKS’ correction module (rxstacks) to make population-based correction of the genotype and haplotype calls did not significantly change the number of unique stacks, polymorphic loci, and SNPs (Tables 2 and 3.)

**Table 3:** Results summary of the STACKS’ population genome analysis using reference approach. Numbers in parenthesis are the results after applying STACKS correction module to make population-based correction.

F2 individual	MseI RARseq				Mse1-Styl RARseq			
	%RARtags mapped to reference	Unique Stacks	Polymorphic Loci	SNPs	%RARtags mapped to reference	Unique Stacks	Polymorphic Loci	SNPs
F2-3	84.97	2874 (2874)	384 (385)	1188 (1190)	83.29	3190 (3190)	513 (517)	1785 (1795)
F2-4	81.65	2134 (2133)	384 (384)	1096 (1094)	80.95	2636 (2635)	480 (488)	1723 (1723)
F2-6	82.65	5111 (5110)	836 (837)	2665 (2643)	86.7	2985 (2984)	477 (481)	1642 (1614)
F2-7	85.34	4068 (4068)	753 (755)	2433 (2440)	84.28	3587 (3587)	723 (732)	2492 (2507)
F2-8	83.82	5149 (5147)	853 (860)	2872 (2858)	85.76	3076 (3076)	572 (581)	2094 (2112)
F2-9	68.5	2626 (2626)	355 (356)	1192 (1193)	61.33	2358 (2358)	407 (410)	1430 (1434)
Average	80.9	3660 (3659.7)	594.2 (596.2)	1907.7 (1903)	80.4	2972 (2971.7)	528.7 (534.8)	1876 (1864)

### Population Heterozygosity and homozygosity in RARseq data

**Fig 2:**
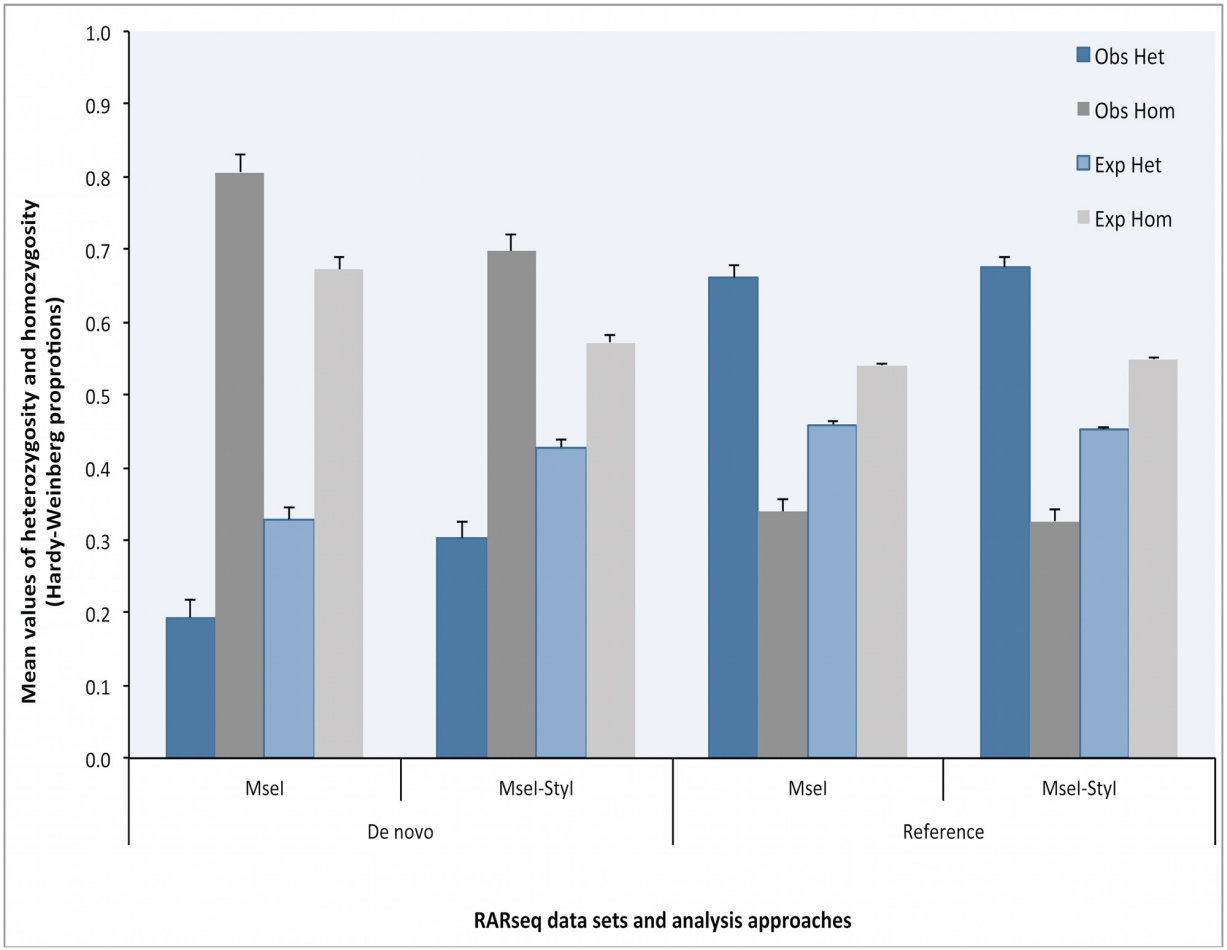
Heterozygozity and homoygozity in RARseq data. Illustration of the observed and expected hetero- and homozygosity calculated from *MseI* and *MseI-Styl* RARseq data using both de novo and reference approaches.

As an indicator of genetic variation, Fig 2 shows the levels of mean heterozygosity and homozygosity as calculated from the allelic frequencies in the test population. The mean expected heterozygosity (H_T_) values over all loci vary from 0.3286 to 0.4594 in both de novo and reference based markers. Similarly, the difference among the means of observed heterozygosity (H_O_) was not significant in de novo and reference based markers (range is 0.1936 to 0.6741.) In the reference-based markers, the mean values of the observed heterozygosity (H_O_) are higher than the expected heterozygosity (H_T_) in both *MseI* and *MseI-StyI* sets. This was true after applying the data correction model as well. This observation is consistent with values expected for an interspecific cross of the parents of a test population or with selection for heterozygosity.

### Inferred Haplotype and gene diversity

**Fig 3:**
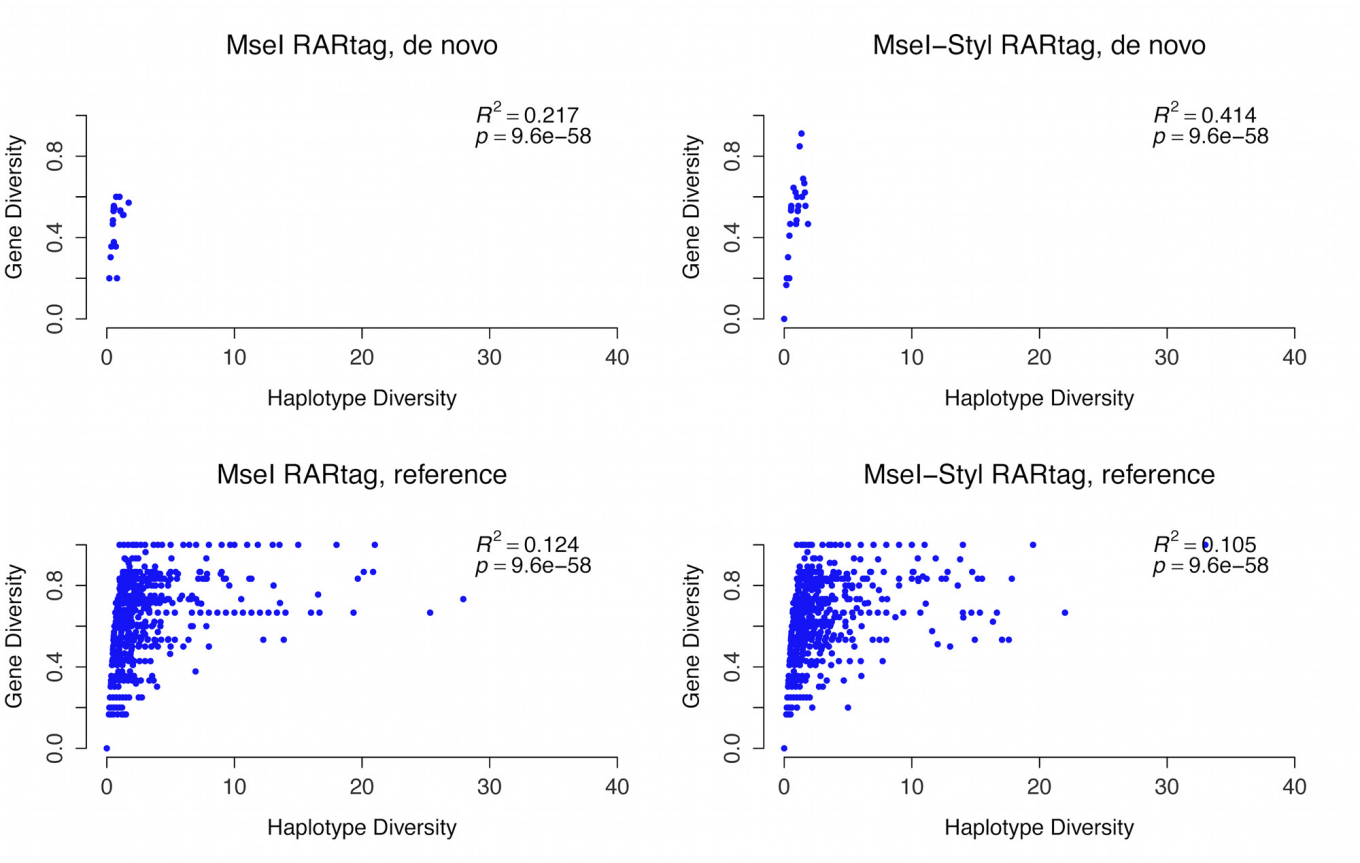
The correlation between haplotype and gene diversity inferred from RARseq data. Both haplotype and gene diversities were calculated from the de novo analysis (top plots) and the reference-based analysis (bottom plots) of *MseI* and *MesI-Styl* RARtags.

The total number of catalog loci (maker loci with sequence coverage in all individuals) with at least one SNP ranges from 345 (ref-based *MseI-Styl*) to 549 (de novo *MseI*). More SNP/allelic loci were detected using the de novo approach compared to the reference approach. The average number of SNPs and alleles per locus are very similar in the four datasets (Table 4.) The number of inferred haplotypes from de novo and reference-based approaches differed dramatically. For instance, out of 549 SNP/allelic loci, only 28 haplotypes were inferred in the de novo *MseI* dataset, whereas 351 haplotypes were inferred from 377 SNP/allelic loci in the reference-based *MseI* dataset (Table 4.) This large difference may be attributed to the pair-wise locus comparison in the de novo analysis, whereas loci are compared to one reference in the reference-based approach. Both haplotype and gene diversity measures were very similar across all datasets (Table 4.) The plot in Fig 3 shows the correlation between mean gene and haplotype diversities in the inferred haplotypes. As evident from the *R* ^2^ and *P* values, there is a weak significant correlation between both gene diversity and haplotype diversity. This could indicate low level of linkage disequilibrium within haplotypes, possibly related to the distribution of expressed genes corresponding to the cDNAs.

**Table 4:** Summary of SNPs, Alleles, Haplotypes, and gene diversity inferred from RARseq data.

	SNPs and Alleles			Haplotype and Gene Diversity			
	Number of catalog loci	Average number of SNP	Average number of alleles	No. Of Haplotypes	Mean Haplotype count	Mean Gene diversity	Mean haplotype diversity
De novo MseI	549	1.767	2.111	28	2.214	0.437	0.686
De novo MseI-Styl	488	1.627	2.113	48	2.271	0.502	0.785
Ref-based MseI	377	1.934	2.228	351	1.912	0.585	0.949
Ref-based MseI-Styl	345	2.235	2.226	321	1.953	0.547	0.892

## Discussion

Genotyping by sequencing (GBS) methods include genome reduction by restriction digestion. However, the amount of DNA after reduction may still be large and yield too much sequence per sample. Many of the identified SNPs are located within non-genic regions, and hence are of lesser functional value. RARseq is a cDNA-based GBS technique that enables the discovery of SNPs and alleles mainly from transcribed regions in the genome. The RARseq technique starts with mRNA transcriptome, which is very small comparing to whole genomes. Constructing RARseq libraries is straightforward and depends greatly on the quality of starting RNA material. High level multiplexing can be attained (in an ongoing experiment, we multiplexed over 210 RARseq libraries). In *silico* digestion of the transcriptome using a variety of restriction enzymes can provide a guide to the expected number of fragments and their length distribution (Table 1). Restriction digestion of double stranded cDNA adds another level of genome reduction and assures balanced representation among samples (Tables 2 and 3.)

Using a test panel, we showed here that RARseq is suitable for population genomic analysis of non-model species using both de novo and reference approaches. To assess RARseq method and evaluate its markers, we compared the de novo and reference-based analyses of *MseI* and *MseI-Styl* RARseq markers. In the reference approach, the parent’s transcriptome is developed and used as a reference. Irrespective of the low sequencing coverage generated in this test, reference based analysis revealed an average of 1907 and 1876 RNA SNPs per individual from single- and dual-digested RARseq data, respectively. Also, the de novo analysis approach revealed over 500 SNPS. When using the whole population and parents, this number of SNP markers should be sufficient to study the population structure and to develop a linkage map.

Giving the inter-specific breeding history of the test panel, we expected high levels of heterozygosity. The polymorphic loci and SNPs detected using de novo approach are much less than those detected using the reference approach. These results are in agreement with the STACKS report on population genomics analysis [11]. As a result, the measured levels of heterozygosity are higher in the reference-based than in the *de novo* approaches (Fig 2.) The difference in the levels of heterozygozity, however, is not statistically significant (H0 Z-test: 0.05312 (*MseI*) and 0.00916 (*MseI-StyI*); P-value: 0.9576 and 0.9927, respectively.) The underestimation of heterozygosity using *de novo* approach could be attributed to the parameters of identifying stacks and detecting SNPs, including mismatches and minimum depth per stack parameters. These parameters could lead to under- or over-merging of alleles, leading to respectively lower or higher levels of polymorphic loci and SNPs. Also, The de novo approach is very sensitive to the depth of coverage in each dataset. Additionally, there are gene duplications and possibly partial genome duplication in Sarracenia [13]. The reference genome approach likely does a better job of sorting out orthologs and paralogs than the de novo approach, at least with the parameters we used. Hence the estimates of heterozygosity are probably more accurate using the reference-based methods.

**Fig 4:**
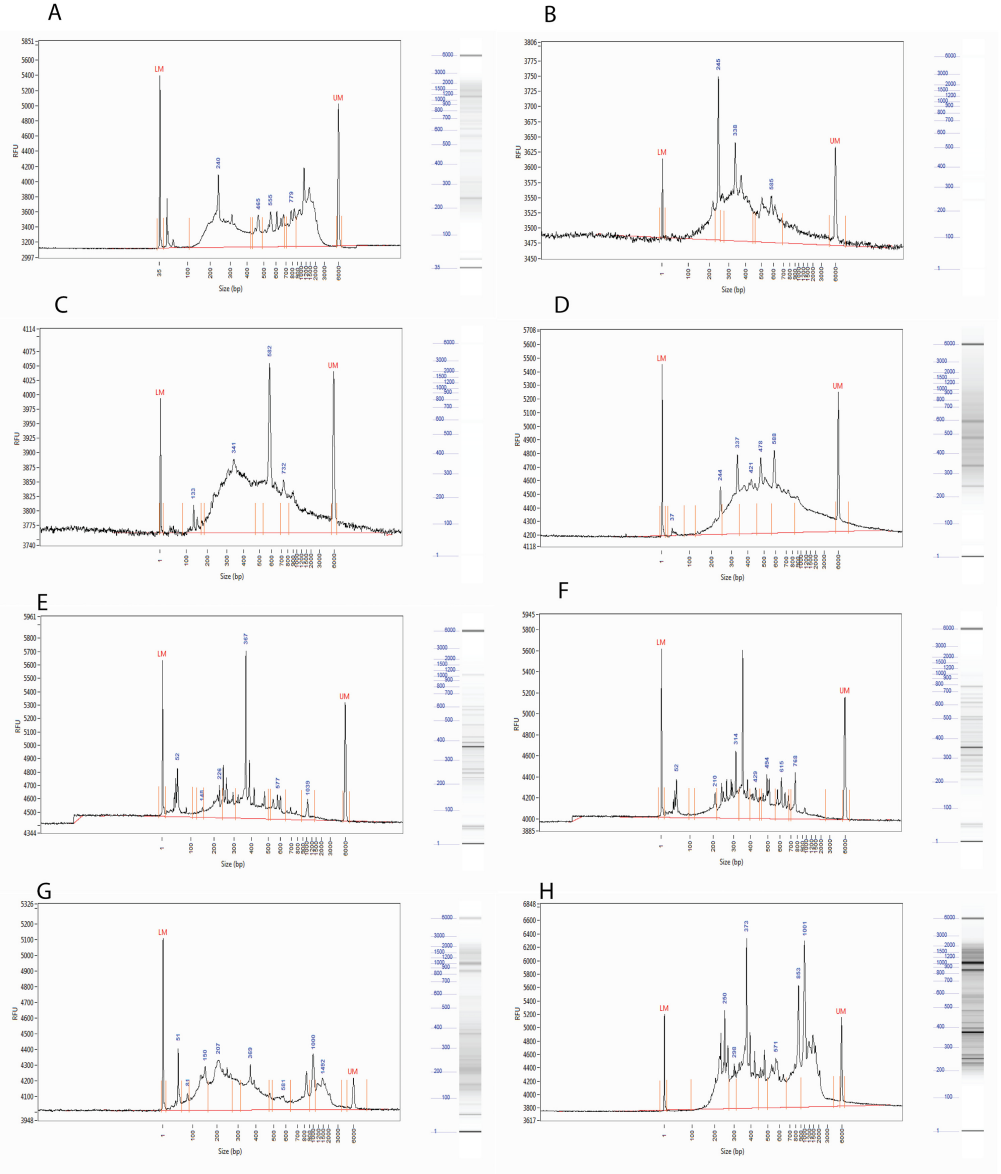
Analysis of the cDNA, digested cDNA, and RARseq libraries using Fragment Analyzer. (A) Double stranded cDNA, (B) *MseI* digested cDNA, (C) *Styl* digested cDNA, (D) *MseI-Styl* double digested cDNA, (E) *MseI* RARseq library, (F) *MseI-Styl* RARseq library, (G) Pool of *MseI* RARseq libraries, (H) Pool of *MseI* RARseq libraries after 1X SPRI cleaning.

In Table 4 and Figs 3 and 4, we summarize the most common measures of genome-wide markers in population genomic analyses: SNPs and alleles, heterozygosity, and haplotype and gene diversity measures. The number of catalog loci is higher in de novo than in reference-based analysis, and higher in *MseI* than *MseI-Styl* data. The average number of SNPs per loci ranged from 1.62 to 2.23, and the average number of inferred alleles per loci is approximately 2 in the four data sets, indicated the robustness of RARseq markers. The distance between markers and their frequency has a profound effect on the haplotype diversity [14]. The inferred haplotype and gene diversity (Fig 3) in this study indicate that the sampled RNA SNP markers might exist in clusters, which acts as blocks with less recombination, hence higher haplotype diversity.

## Conclusion

Our test shows that RARseq protocol is suitable for developing transcriptome-based markers that are suitable for population genomic analysis. In addition to merging both standard RNAseq and GBS protocols, RARseq provides several benefits; including enriching for functional markers, balanced depth of coverage across samples, transcriptome-wide unbiased markers, robustness and it assures sufficient overlap across individuals. This method is an addition to the suite of NGS-based population genomics analysis protocols, such as GBS and RADseq. We foresee this protocol being routinely used in combination with the GBS protocol for population and genetic mapping analyses, especially with species with large genomes and populations.

## Materials and Methods

### Plant material and RNA extraction

F2 plants were grown and directly supervised and managed by the authors in the greenhouse of the Plant Biology Department, University of Georgia. These plants are results of inter-specific genetic crosses performed by the authors; hence these are unique inter-specific genotypes and are not representatives of endangered or protected species. Young un-opened *Sarracenia* leaves were collected removing the leaf near the base of the plant and removing any debris present. If un-opened leaves were not available, the youngest open leaves were taken. These slightly older leaves were sliced open with a clean razor blade and the interior of the leaf washed/cleansed of any debris and insect prey. Leaves were dried and immediately ground to a fine powder under liquid nitrogen. The ground tissue was immediately placed in frozen 15ml tubes and stored at -80°C until RNA extraction. We used a protocol developed for adult pine needles[15], a tissue widely known as difficult with which to work. Instead of washing the RNA pellet with 2M LiCL, we used 90% Ethanol. Total RNA quantity/quality was assessed using 2% TAE gels and NanoDrop 2000 (Thermo Scientific, Wilmington, DE, USA).

### Selection of restriction enzymes for cDNA digestion

We used 454 mRNA transcriptomic sequences generated from *S*. *psittacina* and *S*. *purpurea* to identify the suitable restriction enzymes [13] We used the fuzznuc program from the EMBOSS package[10] to search for the restriction sites patterns in transcriptomes. No mismatches were allowed in the restriction site patterns and target fragment size ranged from 150 to 700bp (Table 1.)

### Test panel

We used a panel of 6 F2 individuals from a cross of *S*. *psittacina* X *S*. *purpurea*. Both single-digested (*MseI*) and dual-digested (*MseI—StyI*) RARseq libraries were prepared from each individual.

### RARseq library preparation

#### 1) cDNA synthesis and cleaning

For the first strand generation, we used RETROscript kit (AM1710) from Life Technologies. We substituted in the Plug-Oligo (5‘-AAGCAGTGGTATCAACGCAGAGTACGGGGG-P-3‘) and CDS-5 RT primer (5'- AAGCAGTGGTATCAACGCAGAGTTTTTGTTTTTTTCTTTTTTTTTTVN -3') (Evrogen JSC., Russia) as they shown to generate more robust and diverse cDNA library. When generating the 2nd strand of cDNA, we used SuperTaq Plus (AM2054) recommended by the RETROscript kit. We followed the manufacturer protocol as following:

**First strand synthesis reaction:** Mix the following reaction ingredients: 2 μg total RNA (may take up to 10 μl of sample), 1μl of 50μM PlugOligo-adapter, 1μl of 50μM CDS-5 adapter, Xμl H20 (complete to 12μl reaction volume). Heat at 72°C for 3min then place on ice for 2min. Add the following: 2μl RT Reaction buffer, 4μl dNTP mix, 1μl RNase Inhibitor, 1μl MMLV-RT (Reverse Transcriptase). Incubate 42–44° for 1 hour. Add 0.4μl of MnCl2 (100mM) Incubate additional 30min.

**Second strand reaction:** Mix the following components: 5μl 1st strand product, 5μl 10X PCR buffer, 4μl of 2.5mM dNTP mix, 2μl of 50μM M1 Primer (5‘-AAGCAGTGGTATCAACGCAGAGT-3‘), 1μl of robust SuperTaq (Life Technologies) DNA polymerase, 33μl H20. Incubate the samples in a Thermo-cycler programed as following: Denaturing at 95°C for 2 min; 16 cycles at 95°C for 15sec, 60°C for 20sec, and 73°C for 3min; and 1 cycle at 66°C for 15sec and 72°C for 3min.

(Notes: cDNA generation completed using RETROscript Kit (AM1710) from Life Technologies. Taq for PCR needs to be Super Taq (Life Technologies), Herculase II (Agilent), or another robust/high fidelity polymerase. Primers/Oligos/Adapters based on Evrogen.)

#### 2) Restriction digestion of double stranded cDNA

We performed both single and double digestion. For single digestion we used the 4-base cutter *MseI* (NEB, R0525S.) For double digestion we used *MseI* and the 6-base cutter, *StyI-HF* (NEB, R3500S.) The digestion reaction steps are as following:

Normalize the cleaned double stranded cDNA to the appropriate concentration (~30ng/μl.) Dilute *MseI* (10U/μl) and *Styl-HF* (20U/μl) enzyme in dilutant A solution (NEB B8001S) to the ratio 1:8 and 1:14, respectively. Set up a 30μl restriction digestion reaction as following:

10μl double-stranded cDNA (a total of 300ng), 3μl Cutsmart buffer (NEB), 1μl diluted *MseI*, 1μl diluted *Styl-HF* (substitute with H20 in case of single digestion), and 15μl H2O. In a Thermocycler, incubate the digestion reaction at 37°C for 4 hours, 65°C for 20 min, and hold at 4°C. Proceed immediately to the next step. The sized distribution pattern of ds-cDNA, *MseI* digested cDNA, Styl digested cDNA, and *MseI-Styl* double digested cDNA are shown in Fig 4A, 4B, 4C and 4D, respectively.

#### 3) Adapter ligation

Prepare the Adapter ligation mix by combining the following components:

30μl digested cDNA from the previous step, 5μl 1.7ng/μl 5’ barcode-adapter, 6μl 280ng/μl 3’ barcode-adapter, 5μl T4 DNA ligase buffer, 1.5μl T4 DNA ligase, and 2μl H2O. The total volume of the ligation reaction mix is 50μl. (Note: we added an excess of the 3’ common adapter because the frequent cutting four-base enzyme will generate a much larger number of fragments than the 6-base cutter enzyme.) Incubate in a Thermocycler at 22°C for 4 hours, 65°C for 20min, and hold at 8°C.

#### 4) Cleaning and size selection

We used the AMPure XP beads (Beckman Coulter Genomics) protocol to remove fragments shorter than 200bp and excess adapters. The cleaning steps are as following:

Add 0.8X volumes (40μl) of a well-mixed AMPure XP beads to the ligation reaction (50μl.) and mix thoroughly by pipetting. Incubate at room temperature for 5min then place the tubes on a magnetic stand and wait for 5min. With the tubes on the magnetic stand, do the following: Remove the clear supernatant. Be careful not to disturb the beads. Add 75μl of freshly prepared 85% EtOH and wait for 2min. Carefully remove the clear EtOH solution. Repeat the ethanol wash steps one more time. Remove the tubes from the magnetic stand and let dry for 4min. (Optional: with 10μl tip, take any excess ethanol might still exist in the tube. Be careful not to touch the bead pellets.) Add 31μl of TE elution buffer and mix thoroughly by pipetting. Incubate at room temperature for 5min. (optional: occasional shaking improves the yield.) Place the tubes on the magnetic stand and wait for 5min, or until the supernatants become very clear. Remove 30μl of the supernatants to new tubes. This constitutes the RARseq libraries. Run 1ul on the Agilent Bioanalyzer using the high sensitivity DNA chips to verify the library size distribution. Take a note of the libraries that might still have short fragments (<200bp.) (Note: running on the libraries on 1.5% Agarose gel with a suitable DNA ladder can be used if a bioanalyzer is not available)

#### 5) PCR enrichment

We used the KAPA HiFi HotStart DNA polymerase (Kapa Biosystems, KK2502) to PCR-enrich each individual library independently to avoid uneven amplification of barcoded libraries. The PCR enrichment steps are as following:

In PCR tubes mix the following: 25μl library from the previous step, 10μl Fidelity buffer (5X), 2μl Forward PCR primer, 2μl Reverse PCR primer, 1.5μl dNTPs mix, 1μl Kapa Hi-Fi Taq polymerase, and 8.5μl H2O. Total reaction volume = 50μl. Incubate the PCR reaction in a thermocycler programmed as following: 1 denaturing cycle: 96°C for 30sec; 8 cycles at 96°C for 30sec, 65°C at 20sec, and 68°C for 25sec; 1 extension cycle at 72°C for 5min; and Final hold at 4°C.

Fig 4E, 4F, and 4G show the size distribution pattern of the PCR enriched libraries of *MesI* RARseq, *MseI-Styl* RARseq, and a pool of *MseI* RARseq libraries, respectively.

#### 6) Library final purification

We used AMPure XP beads to purify the final library fragments and remove excess primers. At the end of step 4, we assessed the library size distribution. At this step, if the library had no short fragment (<200bp), we used 1X volume AMPure beads. If, however, the library contained short fragment we used 0.8X volume AMPure beads. The steps were as following:

Add 1X or 0.8X volumes (50μl and 40μl, respectively) of a well-mixed AMPure XP beads to the PCR reaction (50μl.) and mix thoroughly by pipetting. Incubate at room temperature for 5min then place the tubes on a magnetic stand and wait for 5min. With the tubes on the magnetic stand, do the following: Remove the clear supernatant. Be careful not to disturb the beads. Add 75μl of freshly prepared 85% EtOH and wait for 2min. Carefully remove the clear EtOH solution. Repeat the ethanol wash steps one more time. Remove the tubes from the magnetic stand and let dry for 4min. (Optional: with 10μl tip, take any excess ethanol might still exist in the tube. Be careful not to touch the bead pellets.) Add 31μl of TE elution buffer and mix thoroughly by pipetting. Incubate at room temperature for 5min. (optional: occasional shaking improves the yield.) Place the tubes on the magnetic stand and wait for 5min, or until the supernatants become very clear. Remove 30μl of the supernatants to new tubes. Use 1μl of the library to verify the size distribution on the Agilent High Sensitivity DNA chip (Fig 4H). Quantify the libraries using Qubit 2 fluorometer and Qubit dsDNA HS Assay kit.

#### 7) Pooling

Based on the Qubit-measured concentrations, we normalized the individual libraries then pooled them using equi-molar amounts. The steps are as following:

Normalize each library to 10nM or any appropriate molar concentration. From the equally concentrated libraries, combine equal volumes to make the final library pool. Measure the concentration of the final library pool using Qubit dsDNA HS Assay. Use KAPA Library Quantification qPCR kit to accurately estimate the library pool concentration.

#### 8) Sequencing and coverage

The 12-barcoded libraries were pooled and sequenced on 50% of a Miseq flowcell (Illumina) using the paired-end 250 (PE250) protocol.

### Data analysis

We used the Stacks package [11] to analyze the generated RARseq data. First, reads were processed using Stacks’ *process_tags* module to trim and assign them to corresponding F2 individuals. We then performed population genome analysis using Stacks’ *de novo* and reference approaches.

The parameters for identifying stacks using the de novo approach were: minimum number of raw reads required to create a stack = 3, number of allowed mismatches between loci in single individual = 3, number of mismatches allowed between loci in catalog = 2, minimum number of identical raw reads required to create a stack in progeny individual = 2.

To prepare a reference to be used with the Stack’s reference approach, we assembled the 454 mRNA sequences generated from *S*. *psittacina* and *S*. *purpurea* in one custom transcriptome using CAP3[16]. RARseq reads were mapped to the custom transcriptome using Bowtie2[12]. To identify stacks using the reference approach we used the following parameters: Number of mismatches allowed between loci when building catalog = 0, minimum depth of coverage to report a stack = 3, lower and upper bounds for error rate are 0 and 1 respectively. Chi square significance level required to call a heterozygote or homozygote, alpha = 0.05. Stacks correction parameters: bounded model, confounded limit = 0.25, upper bound for epsilon (error rate) = 0.1, chi square significance level (alpha) = 0.05, minimum log likelihood required to keep a catalog locus = -10, and prune out non-biological haplotypes unlikely to occur in the population.

Nanodrop to establish volumes required for pooling (i.e., after all of these steps, digestion, ligation, ampure beads, PCR, individuals are going to be at different concentrations, this is the last chance to fix this. Desired concentrations 30–100ng/ul.
